# Ethyl 4-{2,6-dichloro-4-[3-(2,6-difluoro­benzo­yl)ureido]phen­oxy}butanoate

**DOI:** 10.1107/S1600536808024987

**Published:** 2008-08-09

**Authors:** Yin-Hong Liu, Fang-Shi Li, Yi Li, Da-Sheng Yu, Chui Lu

**Affiliations:** a Department of Applied Chemistry, College of Science, Nanjing University of Technolgy, Xinmofan Road No. 5, Nanjing 210009, People’s Republic of China

## Abstract

The title compound, C_20_H_18_Cl_2_F_2_N_2_O_5_, is considered to belong to the fourth generation of insecticides with properties such as high selectivity, low acute toxicity for mammals and high biological activity. An intramolecular N—H⋯O hydrogen bond results in the formation of a six-membered ring. In the crystal structure, intermolecular N—H⋯O and C—H⋯F hydrogen bonds link the molecules.

## Related literature

For related literature, see: Wang *et al.* (1998 ▶, 1999 ▶). For bond-length data, see: Allen *et al.* (1987 ▶).
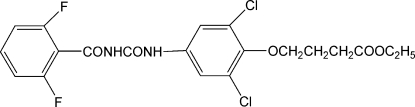


## Experimental

### 

#### Crystal data

C_20_H_18_Cl_2_F_2_N_2_O_5_
*M* *_r_* = 475.26
Monoclinic, 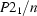

*a* = 11.262 (2) Å
*b* = 10.463 (2) Å
*c* = 18.613 (4) Å
β = 98.78 (3)°
*V* = 2167.5 (8) Å^3^
*Z* = 4
Mo *K*α radiation
μ = 0.35 mm^−1^
*T* = 293 (2) K
0.40 × 0.30 × 0.20 mm

#### Data collection

Enraf–Nonius CAD-4 diffractometer
Absorption correction: ψ scan (North *et al.*, 1968 ▶) *T*_min_ = 0.872, *T*_max_ = 0.933
4157 measured reflections
3944 independent reflections
2385 reflections with *I* > 2σ(*I*)
*R*_int_ = 0.041
3 standard reflections every 200 reflections intensity decay: none

#### Refinement

*R*[*F* ^2^ > 2σ(*F* ^2^)] = 0.082
*wR*(*F* ^2^) = 0.290
*S* = 1.13
3944 reflections
268 parameters
H-atom parameters constrained
Δρ_max_ = 0.77 e Å^−3^
Δρ_min_ = −0.99 e Å^−3^

### 

Data collection: *CAD-4 Software* (Enraf–Nonius, 1989 ▶); cell refinement: *CAD-4 Software*; data reduction: *XCAD4* (Harms & Wocadlo, 1995 ▶); program(s) used to solve structure: *SHELXS97* (Sheldrick, 2008 ▶); program(s) used to refine structure: *SHELXL97* (Sheldrick, 2008 ▶); molecular graphics: *SHELXL97*; software used to prepare material for publication: *SHELXL97*.

## Supplementary Material

Crystal structure: contains datablocks I, global. DOI: 10.1107/S1600536808024987/cs2088sup1.cif

Structure factors: contains datablocks I. DOI: 10.1107/S1600536808024987/cs2088Isup2.hkl

Additional supplementary materials:  crystallographic information; 3D view; checkCIF report

## Figures and Tables

**Table 1:** Hydrogen-bond geometry (Å, °)

*D*—H⋯*A*	*D*—H	H⋯*A*	*D*⋯*A*	*D*—H⋯*A*
N2—H2*A*⋯O4^i^	0.86	2.00	2.856 (6)	173
C5—H5*A*⋯F2^ii^	0.97	2.44	3.201 (8)	135
N1—H1*A*⋯O5	0.86	1.97	2.675 (7)	138

Symmetry codes: (i) 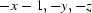; (ii) 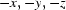.

**Fig. 1.**
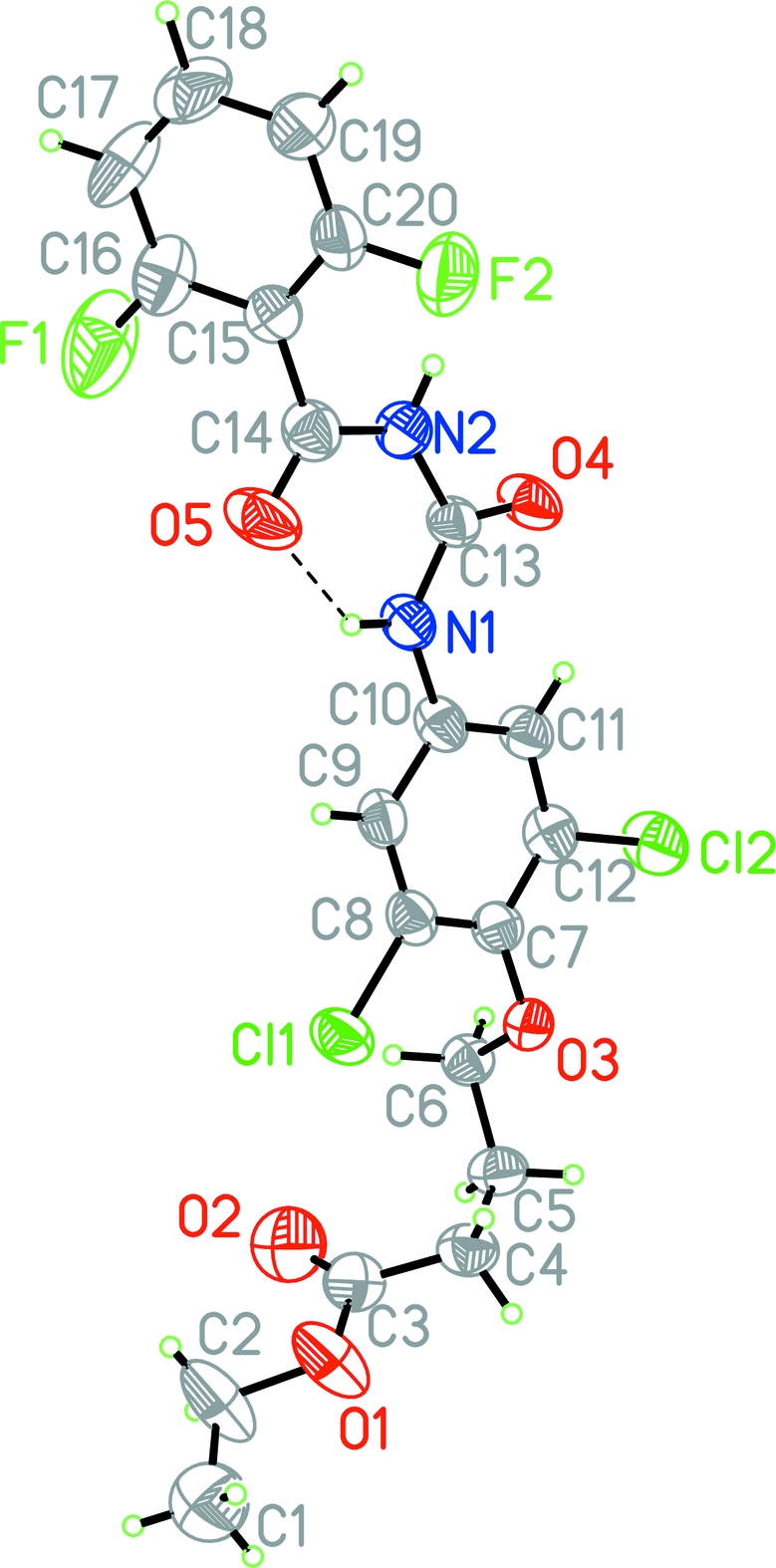
The molecular structure of the title compound with the atom-numbering scheme. Displacement ellipsoids are drawn at the 50% probability level. N—H···O intramolecular hydrogen bond is shown by dashed line.

**Fig. 2.**
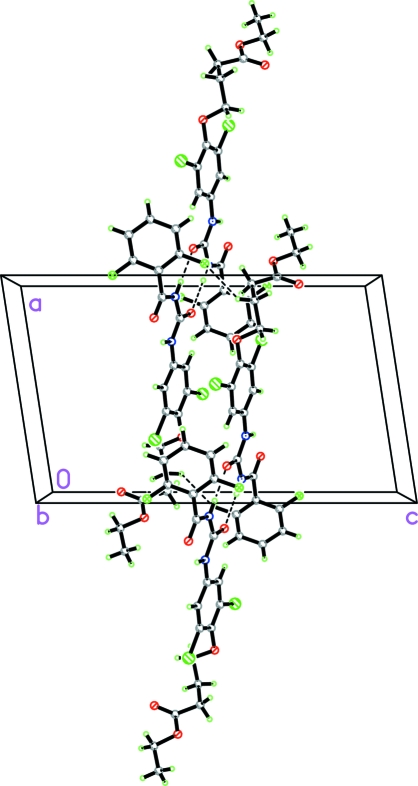
A packing diagram detail from the crystal structure. N—H···O and C—H···F intermolecular hydrogen bonds are shown by dashed lines.

## supplementary crystallographic information

### Comment

The title compound is generally recognized as an insect growth regulator that interferes with chitin synthesis in target pests, causing death or abortive development (Wang *et al.* 1998). Bonding dimensions conform to expected values (Allen *et al.*, 1987).

### Experimental

The title compound was prepared according to a literature method (Wang *et al.* 1999). The crystals suitable for X-ray analysis were obtained by dissolving it (0.1 g) in acetonitrile (25 ml) and evaporating the solvent slowly at room temperature for about 6 d.

### Refinement

H atoms were positioned geometrically, with C—H distances of 0.93 and of 0.97 Å for aromatic and methyl H atoms and with N—H = 0.86 Å for amido H atoms. All H atoms were constrained to ride on their parent atoms with *U*_iso_(H) = *xU*_eq_(C,*N*), where *x* = 1.2 for all H atoms.

### Crystal data

**Table:**

C_20_H_18_Cl_2_F_2_N_2_O_5_	*F*_000_ = 976
*M**_r_* = 475.26	*D*_x_ = 1.456 Mg m^−^^3^
Monoclinic, *P*2_1_/*n*	Mo *K*α radiation λ = 0.71073 Å
Hall symbol: -P 2yn	Cell parameters from 25 reflections
*a* = 11.262 (2) Å	θ = 9–12º
*b* = 10.463 (2) Å	µ = 0.35 mm^−^^1^
*c* = 18.613 (4) Å	*T* = 293 (2) K
β = 98.78 (3)º	Block, colourless
*V* = 2167.5 (8) Å^3^	0.40 × 0.30 × 0.20 mm
*Z* = 4	

### Data collection

**Table:**

Enraf–Nonius CAD-4 diffractometer	*R*_int_ = 0.041
Radiation source: fine-focus sealed tube	θ_max_ = 25.3º
Monochromator: graphite	θ_min_ = 2.0º
*T* = 293(2) K	*h* = −13→13
ω/2θ scans	*k* = 0→12
Absorption correction: ψ scan(North *et al.*, 1968)	*l* = 0→22
*T*_min_ = 0.872, *T*_max_ = 0.933	3 standard reflections
4157 measured reflections	every 200 reflections
3944 independent reflections	intensity decay: none
2385 reflections with *I* > 2σ(*I*)	

### Refinement

**Table:**

Refinement on *F*^2^	Secondary atom site location: difference Fourier map
Least-squares matrix: full	Hydrogen site location: inferred from neighbouring sites
*R*[*F*^2^ > 2σ(*F*^2^)] = 0.082	H-atom parameters constrained
*wR*(*F*^2^) = 0.290	*w* = 1/[σ^2^(*F*_o_^2^) + (0.1372*P*)^2^ + 4.2262*P*] where *P* = (*F*_o_^2^ + 2*F*_c_^2^)/3
*S* = 1.13	(Δ/σ)_max_ < 0.001
3944 reflections	Δρ_max_ = 0.77 e Å^−^^3^
268 parameters	Δρ_min_ = −0.99 e Å^−^^3^
Primary atom site location: structure-invariant direct methods	Extinction correction: none

### Special details

**Table:**

Experimental. (North *et al.*, 1968)

### Fractional atomic coordinates and isotropic or equivalent isotropic displacement parameters (Å^2^)

**Table:**

	*x*	*y*	*z*	*U*_iso_*/*U*_eq_	
Cl1	0.23061 (14)	0.09615 (16)	0.16895 (10)	0.0611 (5)	
Cl2	0.02268 (15)	−0.30602 (15)	0.02019 (10)	0.0672 (6)	
F1	−0.5092 (6)	0.3204 (6)	0.2109 (3)	0.127 (2)	
F2	−0.5604 (4)	0.3418 (6)	−0.0403 (2)	0.0995 (17)	
N1	−0.2177 (4)	0.0877 (5)	0.0826 (3)	0.0473 (12)	
H1A	−0.2132	0.1606	0.1042	0.057*	
O1	0.5929 (5)	0.0111 (5)	0.2331 (3)	0.0813 (16)	
C1	0.7375 (8)	0.1501 (10)	0.2970 (5)	0.095	
H1B	0.7622	0.1938	0.3421	0.142*	
H1C	0.7173	0.2116	0.2588	0.142*	
H1D	0.8019	0.0969	0.2861	0.142*	
N2	−0.4207 (4)	0.1420 (5)	0.0532 (3)	0.0481 (12)	
H2A	−0.4908	0.1202	0.0316	0.058*	
O2	0.4801 (5)	−0.1199 (6)	0.2895 (3)	0.092	
C2	0.6371 (8)	0.0743 (9)	0.3027 (4)	0.092 (3)	
H2B	0.5738	0.1271	0.3170	0.110*	
H2C	0.6583	0.0100	0.3400	0.110*	
O3	0.2254 (3)	−0.1568 (4)	0.09703 (19)	0.0458 (10)	
C3	0.5158 (6)	−0.0854 (7)	0.2341 (3)	0.0544 (16)	
O4	−0.3529 (4)	−0.0477 (4)	0.0170 (2)	0.0578 (12)	
C4	0.4795 (5)	−0.1407 (6)	0.1610 (4)	0.0545 (16)	
H4A	0.5512	−0.1638	0.1411	0.065*	
H4B	0.4376	−0.0757	0.1297	0.065*	
O5	−0.3196 (4)	0.3054 (5)	0.1175 (3)	0.0775 (16)	
C5	0.3991 (5)	−0.2578 (6)	0.1597 (4)	0.0513 (15)	
H5A	0.4037	−0.3058	0.1156	0.062*	
H5B	0.4293	−0.3121	0.2006	0.062*	
C6	0.2705 (5)	−0.2267 (6)	0.1628 (3)	0.0436 (13)	
H6A	0.2246	−0.3046	0.1655	0.052*	
H6B	0.2640	−0.1751	0.2053	0.052*	
C7	0.1154 (5)	−0.1029 (6)	0.0964 (3)	0.0419 (13)	
C8	0.1028 (5)	0.0189 (6)	0.1269 (3)	0.0431 (13)	
C9	−0.0055 (5)	0.0791 (6)	0.1230 (3)	0.0455 (14)	
H9A	−0.0108	0.1591	0.1440	0.055*	
C10	−0.1091 (5)	0.0182 (5)	0.0867 (3)	0.0440 (14)	
C11	−0.1009 (5)	−0.1023 (5)	0.0560 (3)	0.0453 (14)	
H11A	−0.1688	−0.1437	0.0323	0.054*	
C12	0.0113 (5)	−0.1586 (5)	0.0618 (3)	0.0464 (14)	
C13	−0.3273 (5)	0.0529 (6)	0.0489 (3)	0.0428 (13)	
C14	−0.4138 (6)	0.2591 (6)	0.0874 (3)	0.0509 (15)	
C15	−0.5308 (5)	0.3250 (5)	0.0855 (3)	0.0431 (13)	
C16	−0.5738 (7)	0.3548 (7)	0.1491 (4)	0.0622 (18)	
C17	−0.6834 (8)	0.4151 (7)	0.1493 (5)	0.076 (2)	
H17A	−0.7108	0.4325	0.1930	0.091*	
C18	−0.7493 (7)	0.4481 (7)	0.0858 (5)	0.074 (2)	
H18A	−0.8236	0.4871	0.0854	0.089*	
C19	−0.7083 (6)	0.4251 (7)	0.0223 (4)	0.071 (2)	
H19A	−0.7535	0.4498	−0.0215	0.085*	
C20	−0.6010 (6)	0.3657 (7)	0.0230 (3)	0.0574 (17)	

### Atomic displacement parameters (Å^2^)

**Table:**

	*U*^11^	*U*^22^	*U*^33^	*U*^12^	*U*^13^	*U*^23^
Cl1	0.0473 (9)	0.0581 (10)	0.0746 (11)	−0.0088 (7)	−0.0015 (7)	−0.0205 (8)
Cl2	0.0649 (11)	0.0426 (9)	0.0892 (13)	0.0079 (8)	−0.0042 (9)	−0.0222 (8)
F1	0.181 (6)	0.146 (5)	0.057 (3)	0.081 (5)	0.027 (3)	0.020 (3)
F2	0.104 (3)	0.145 (5)	0.048 (2)	0.052 (3)	0.006 (2)	−0.015 (3)
N1	0.047 (3)	0.041 (3)	0.050 (3)	0.010 (2)	−0.007 (2)	−0.010 (2)
O1	0.092 (4)	0.072 (3)	0.068 (3)	−0.033 (3)	−0.024 (3)	0.011 (3)
C1	0.095	0.095	0.095	0.000	0.014	0.000
N2	0.048 (3)	0.038 (3)	0.056 (3)	0.009 (2)	0.001 (2)	−0.010 (2)
O2	0.092	0.092	0.092	0.000	0.014	0.000
C2	0.109 (7)	0.085 (6)	0.068 (5)	−0.039 (5)	−0.029 (5)	−0.001 (4)
O3	0.042 (2)	0.058 (3)	0.038 (2)	0.0023 (19)	0.0072 (16)	−0.0027 (18)
C3	0.055 (4)	0.060 (4)	0.047 (4)	−0.001 (3)	0.006 (3)	0.015 (3)
O4	0.048 (2)	0.041 (2)	0.077 (3)	0.0065 (19)	−0.015 (2)	−0.021 (2)
C4	0.045 (3)	0.056 (4)	0.063 (4)	−0.008 (3)	0.009 (3)	−0.001 (3)
O5	0.053 (3)	0.058 (3)	0.111 (4)	0.000 (2)	−0.020 (3)	−0.034 (3)
C5	0.038 (3)	0.052 (4)	0.063 (4)	0.002 (3)	0.008 (3)	−0.001 (3)
C6	0.043 (3)	0.042 (3)	0.045 (3)	0.003 (3)	0.004 (2)	0.001 (3)
C7	0.041 (3)	0.048 (3)	0.036 (3)	0.002 (3)	0.004 (2)	0.004 (3)
C8	0.046 (3)	0.043 (3)	0.040 (3)	−0.009 (3)	0.004 (2)	−0.003 (3)
C9	0.052 (3)	0.043 (3)	0.039 (3)	0.002 (3)	0.001 (3)	−0.008 (3)
C10	0.047 (3)	0.040 (3)	0.041 (3)	0.002 (3)	−0.004 (2)	−0.001 (3)
C11	0.045 (3)	0.038 (3)	0.051 (3)	0.002 (3)	−0.001 (3)	−0.006 (3)
C12	0.054 (3)	0.035 (3)	0.049 (3)	0.001 (3)	0.005 (3)	0.000 (3)
C13	0.040 (3)	0.043 (3)	0.043 (3)	0.011 (3)	−0.004 (2)	−0.003 (3)
C14	0.053 (4)	0.046 (4)	0.051 (4)	0.001 (3)	0.003 (3)	−0.002 (3)
C15	0.044 (3)	0.037 (3)	0.049 (3)	−0.005 (2)	0.008 (3)	−0.001 (3)
C16	0.091 (5)	0.051 (4)	0.049 (4)	0.010 (4)	0.024 (4)	0.011 (3)
C17	0.101 (6)	0.055 (4)	0.085 (6)	0.026 (4)	0.056 (5)	0.005 (4)
C18	0.060 (4)	0.050 (4)	0.114 (7)	0.015 (3)	0.020 (4)	−0.022 (4)
C19	0.062 (4)	0.073 (5)	0.074 (5)	0.016 (4)	0.001 (4)	−0.019 (4)
C20	0.059 (4)	0.061 (4)	0.050 (4)	0.007 (3)	−0.002 (3)	−0.018 (3)

### Geometric parameters (Å, °)

**Table:**

Cl1—C8	1.730 (6)	C4—H4B	0.9700
Cl2—C12	1.739 (6)	O5—C14	1.222 (7)
F1—C16	1.314 (9)	C5—C6	1.494 (8)
F2—C20	1.350 (7)	C5—H5A	0.9700
N1—C13	1.347 (7)	C5—H5B	0.9700
N1—C10	1.414 (7)	C6—H6A	0.9700
N1—H1A	0.8600	C6—H6B	0.9700
O1—C3	1.333 (8)	C7—C12	1.379 (8)
O1—C2	1.472 (9)	C7—C8	1.410 (8)
C1—C2	1.399 (11)	C8—C9	1.365 (8)
C1—H1B	0.9600	C9—C10	1.408 (8)
C1—H1C	0.9600	C9—H9A	0.9300
C1—H1D	0.9600	C10—C11	1.394 (8)
N2—C14	1.378 (8)	C11—C12	1.383 (8)
N2—C13	1.417 (7)	C11—H11A	0.9300
N2—H2A	0.8600	C14—C15	1.482 (8)
O2—C3	1.217 (8)	C15—C20	1.372 (9)
C2—H2B	0.9700	C15—C16	1.381 (8)
C2—H2C	0.9700	C16—C17	1.386 (10)
O3—C7	1.360 (6)	C17—C18	1.342 (11)
O3—C6	1.449 (7)	C17—H17A	0.9300
C3—C4	1.477 (9)	C18—C19	1.354 (10)
O4—C13	1.223 (7)	C18—H18A	0.9300
C4—C5	1.522 (9)	C19—C20	1.358 (9)
C4—H4A	0.9700	C19—H19A	0.9300
			
C13—N1—C10	127.6 (5)	O3—C7—C8	121.3 (5)
C13—N1—H1A	116.2	C12—C7—C8	116.1 (5)
C10—N1—H1A	116.2	C9—C8—C7	122.6 (5)
C3—O1—C2	117.4 (6)	C9—C8—Cl1	118.9 (4)
C2—C1—H1B	109.5	C7—C8—Cl1	118.4 (4)
C2—C1—H1C	109.5	C8—C9—C10	119.1 (5)
H1B—C1—H1C	109.5	C8—C9—H9A	120.5
C2—C1—H1D	109.5	C10—C9—H9A	120.5
H1B—C1—H1D	109.5	C11—C10—C9	120.2 (5)
H1C—C1—H1D	109.5	C11—C10—N1	123.7 (5)
C14—N2—C13	128.4 (5)	C9—C10—N1	116.1 (5)
C14—N2—H2A	115.8	C12—C11—C10	118.1 (5)
C13—N2—H2A	115.8	C12—C11—H11A	121.0
C1—C2—O1	110.9 (8)	C10—C11—H11A	121.0
C1—C2—H2B	109.5	C7—C12—C11	123.9 (5)
O1—C2—H2B	109.5	C7—C12—Cl2	117.9 (5)
C1—C2—H2C	109.5	C11—C12—Cl2	118.1 (5)
O1—C2—H2C	109.5	O4—C13—N1	126.2 (5)
H2B—C2—H2C	108.1	O4—C13—N2	118.2 (5)
C7—O3—C6	114.7 (4)	N1—C13—N2	115.6 (5)
O2—C3—O1	122.5 (7)	O5—C14—N2	123.3 (6)
O2—C3—C4	125.6 (6)	O5—C14—C15	122.2 (6)
O1—C3—C4	111.9 (5)	N2—C14—C15	114.5 (5)
C3—C4—C5	114.3 (6)	C20—C15—C16	115.1 (6)
C3—C4—H4A	108.7	C20—C15—C14	124.0 (5)
C5—C4—H4A	108.7	C16—C15—C14	120.8 (6)
C3—C4—H4B	108.7	F1—C16—C15	117.9 (6)
C5—C4—H4B	108.7	F1—C16—C17	119.7 (6)
H4A—C4—H4B	107.6	C15—C16—C17	122.3 (7)
C6—C5—C4	113.7 (5)	C18—C17—C16	119.2 (7)
C6—C5—H5A	108.8	C18—C17—H17A	120.4
C4—C5—H5A	108.8	C16—C17—H17A	120.4
C6—C5—H5B	108.8	C17—C18—C19	120.4 (7)
C4—C5—H5B	108.8	C17—C18—H18A	119.8
H5A—C5—H5B	107.7	C19—C18—H18A	119.8
O3—C6—C5	107.1 (5)	C18—C19—C20	119.7 (7)
O3—C6—H6A	110.3	C18—C19—H19A	120.1
C5—C6—H6A	110.3	C20—C19—H19A	120.1
O3—C6—H6B	110.3	F2—C20—C19	119.8 (6)
C5—C6—H6B	110.3	F2—C20—C15	117.1 (6)
H6A—C6—H6B	108.5	C19—C20—C15	123.2 (6)
O3—C7—C12	122.4 (5)		
			
C3—O1—C2—C1	165.2 (8)	C10—C11—C12—C7	0.4 (9)
C2—O1—C3—O2	1.5 (10)	C10—C11—C12—Cl2	−177.4 (4)
C2—O1—C3—C4	−179.5 (7)	C10—N1—C13—O4	2.0 (10)
O2—C3—C4—C5	−5.6 (10)	C10—N1—C13—N2	−179.6 (5)
O1—C3—C4—C5	175.5 (5)	C14—N2—C13—O4	178.4 (6)
C3—C4—C5—C6	79.5 (7)	C14—N2—C13—N1	−0.2 (9)
C7—O3—C6—C5	−169.7 (5)	C13—N2—C14—O5	3.4 (10)
C4—C5—C6—O3	64.3 (7)	C13—N2—C14—C15	−176.1 (5)
C6—O3—C7—C12	−99.1 (6)	O5—C14—C15—C20	116.7 (8)
C6—O3—C7—C8	85.8 (6)	N2—C14—C15—C20	−63.8 (8)
O3—C7—C8—C9	175.6 (5)	O5—C14—C15—C16	−60.2 (9)
C12—C7—C8—C9	0.3 (8)	N2—C14—C15—C16	119.3 (6)
O3—C7—C8—Cl1	−2.8 (7)	C20—C15—C16—F1	−178.7 (7)
C12—C7—C8—Cl1	−178.2 (4)	C14—C15—C16—F1	−1.5 (10)
C7—C8—C9—C10	−0.5 (9)	C20—C15—C16—C17	3.5 (10)
Cl1—C8—C9—C10	177.9 (4)	C14—C15—C16—C17	−179.3 (6)
C8—C9—C10—C11	0.7 (9)	F1—C16—C17—C18	−179.1 (8)
C8—C9—C10—N1	−178.0 (5)	C15—C16—C17—C18	−1.3 (12)
C13—N1—C10—C11	−1.2 (9)	C16—C17—C18—C19	−1.3 (12)
C13—N1—C10—C9	177.4 (6)	C17—C18—C19—C20	1.4 (12)
C9—C10—C11—C12	−0.6 (9)	C18—C19—C20—F2	179.5 (7)
N1—C10—C11—C12	178.0 (5)	C18—C19—C20—C15	1.1 (11)
O3—C7—C12—C11	−175.5 (5)	C16—C15—C20—F2	178.1 (6)
C8—C7—C12—C11	−0.2 (9)	C14—C15—C20—F2	1.0 (10)
O3—C7—C12—Cl2	2.2 (8)	C16—C15—C20—C19	−3.4 (10)
C8—C7—C12—Cl2	177.5 (4)	C14—C15—C20—C19	179.5 (6)

### Hydrogen-bond geometry (Å, °)

**Table:**

*D*—H···*A*	*D*—H	H···*A*	*D*···*A*	*D*—H···*A*
N2—H2A···O4^i^	0.86	2.00	2.856 (6)	173
C5—H5A···F2^ii^	0.97	2.44	3.201 (8)	135
N1—H1A···O5	0.86	1.97	2.675 (7)	138

Symmetry codes: (i) −*x*−1, −*y*, −*z*; (ii) −*x*, −*y*, −*z*.
